# Perceptions of the COVID-19 vaccine among patients with cancer: a single-institution survey

**DOI:** 10.2217/fon-2021-0265

**Published:** 2021-08-02

**Authors:** Elissar Moujaess, Naji Bou Zeid, Ramy Samaha, Joud Sawan, Hampig Kourie, Chris Labaki, Roy Chebel, Georges Chahine, Fadi El Karak, Fadi Nasr, Marwan Ghosn, Jad Wakim, Joseph Kattan

**Affiliations:** ^1^Department of Hematology-Oncology, Hotel-Dieu de France University Hospital, Saint Joseph University, Beirut, Lebanon

**Keywords:** acceptance, cancer, COVID-19, hesitancy, survey, vaccine

## Abstract

**Aims:** This paper reports the results of a survey assessing the acceptance of the coronavirus disease 2019 (COVID-19) vaccine among patients with cancer. **Patients and methods:** In total, 111 adult patients with cancer from a single institution were asked to complete a questionnaire designed to assess their knowledge about the vaccine, their readiness to be vaccinated and the determinants of their decision. **Results:** 61.3% of the patients considered themselves more vulnerable to COVID-19 than the general population. Television, radio and newspapers were the major sources of information about the vaccine. A total of 55% of the patients were ready to be vaccinated and 14.4% refused the vaccine. The main reason for refusal was incompatibility with patients' disease or treatment. **Conclusion:** Most of the patients in this institutional sample accepted the COVID-19 vaccine. Better communication of information with patients is needed to decrease vaccine hesitancy.

## Lay abstract

Major cancer societies consider vaccinating patients with cancer against coronavirus disease 2019 (COVID-19) a priority. The investigators conducted a survey assessing perceptions of the vaccine among patients with cancer. A total of 111 patients were asked to complete a questionnaire evaluating their knowledge about the vaccine, their readiness to be vaccinated and the determinants of their decision. Most (61.3%) patients considered themselves more susceptible to COVID-19 than the general population. Television, radio and newspapers were the major sources of information about the vaccine. The majority of patients (55%) were ready to be vaccinated and 14.4% refused the vaccine. The main reason for refusal was incompatibility with patients' disease or treatment. Better communication with patients is needed to decrease vaccine hesitancy.

At the end of 2019, a novel coronavirus called coronavirus disease 2019 (COVID-19) or severe acute respiratory syndrome coronavirus 2 (SARS-CoV-2), caused a cluster of pneumonia cases in Wuhan, China. The virus rapidly spread worldwide and was declared a public health emergency of international concern on January 30, 2020, then a pandemic in March 2020, by the WHO [1]. Since then, managing patients with cancer during the pandemic has been problematic [2]. Practitioners are often condemned to choose between reducing patients' exposure to the virus in healthcare centers at the expense of delaying testing and treatment; or proceeding with timely medical or surgical management regardless of the risk of contracting COVID-19 and the potential complications on an immunocompromised host. Not only are physicians confronted with this dilemma, but patients also have this dual fear.

There is limited experimental data on the effect of COVID-19 on the health of patients with cancer. It is now established that some biomarkers are predictive of a worse outcome in patients with a COVID-19 infection, such as high cytokine levels (IL-6, TNF-α and others), high D-dimer levels, lymphocytopenia and neutropenia. It is also presumed that patients with metabolic syndrome, who already have a high level of the previously cited markers of inflammation, have a worse prognosis and a higher risk of cytokine storm when infected with COVID-19. Since patients with cancer also have high levels of inflammation markers and D-dimer and can often have lymphocytopenia or neutropenia, the fear of critical or even fatal consequences of a COVID-19 infection on patients with cancer is justified [3].

To date, while COVID-19-related mortality is still increasing in many countries and no specific treatment has been highly recommended, vaccination to prevent the infection is the most promising strategy for flattening the curve. Almost a year after the outbreak surge, the first emergency use authorization (EUA) was granted by the US FDA on December 11, 2020, for the Pfizer-BioNTech COVID-19 mRNA vaccine BNT162b2 [4], and on December 18, 2020 for the Moderna mRNA vaccine mRNA-1273, based on two randomized controlled trials [5]. Both vaccines were deemed safe with few severe adverse events, and with mortality rates not exceeding those in the placebo arm in both trials [6,7]. Data concerning vaccine safety and efficacy in immunocompromised patients, including patients with cancer, are insufficient. Approximately 4% of the patients participating in the BNT162b2 vaccine phase III trial had a malignancy [6], and the mRNA-1273 vaccine phase III trial excluded patients with an immunocompromised state or who had recently received an immune suppressive therapy. One patient with chronic lymphocytic leukemia was included and died after receiving the injection, but was randomized to the placebo group [7]. Unlike classical live or attenuated virus vaccines, mRNA vaccines trigger an immune response by translating the viral RNA into a spike protein expressed by the virus [8]. This suggests that mRNA vaccines do not share the same risk profile for immunocompromised hosts.

According to the interim guidelines from the US CDC Advisory Committee on Immunization Practices (ACIP) on COVID-19 vaccination, immunocompromised patients are more vulnerable to COVID-19 complications and may receive COVID-19 vaccination if they have no contraindications to any component of the specific vaccine, but should be counseled about the unknown vaccine safety profile and effectiveness in immunocompromised populations, as well as the potential for insufficient immune responses [9]. This concerns not only patients with cancer but all patients with immunocompromised states, such as HIV and other conditions. Guidelines from cancer societies are now available and recommend vaccination despite the paucity of data in this specific population. In fact, the American Society of Clinical Oncology (ASCO) and Infectious Diseases Society of America (IDSA) discussed the importance of COVID-19 vaccination during a webinar and concluded that patients with active cancer and cancer survivors may receive mRNA vaccines if they have no contraindication. Experts emphasized that although immunocompromised patients may experience a decreased response to the vaccine, it may still confer some benefit in reducing the risk or severity of COVID-19 in patients with cancer [10]. The European Society for Medical Oncology (ESMO) also released similar statements on vaccination against COVID-19, encouraging vaccination of all patients with cancer and giving priority to patients with active disease or those receiving anticancer treatment where the resources are limited [11]. These guidelines are also consistent with the preliminary recommendations of the National Comprehensive Cancer Network (NCCN) COVID-19 Vaccination Advisory Committee, recommending that patients with cancer be given an approved vaccine as soon as it becomes available, except for hematopoietic stem cell recipients and patients treated with chimeric antigen receptor-modified T-cell therapy (CAR-T) who should have their vaccination delayed [12].

Despite the availability of clear recommendations, the authors noticed that patients with cancer in Hotel-Dieu de France, a tertiary university hospital in Beirut, Lebanon, have many concerns regarding COVID-19 vaccination. In light of this observation, a simple survey was conducted to assess patients' knowledge about the vaccine, their readiness to be vaccinated and the main determinants that drive their decision.

## Patients & methods

### Patients & ethical considerations

Patients were selected from the same day ward of the Department of Hematology and Oncology of Hotel-Dieu de France University Hospital, a tertiary hospital in Beirut, Lebanon. They were interviewed between January 25, 2021, and February 12, 2021. The first batch of vaccine was scheduled to arrive in Lebanon on the 14th of February 2021, and recruitment ended before this date. Patients included in the study were adults (≥18 years) with any solid or hematologic malignancy and any disease stage, receiving chemotherapy, targeted therapy, immunotherapy or supportive care (such as transfusion for anemia related to malignancy, or bisphosphonates for skeletal-related events from anticancer therapies). Patients on hormonotherapy who visited the same day ward for other concomitant therapies were also included. Patients receiving therapies or transfusion for benign hematologic disorders were excluded. Patients for whom linguistic hindrances preventing completion of the questionnaire were also excluded.

The study was approved by the Saint Joseph University Ethics committee. During recruitment, the investigator explained to the patients that their identity would be strictly confidential. Every patient for his/her agreement to participate in the survey. After accepting, the patient signed a form in Arabic, declaring informed consent. The interview was conducted privately and without interruption from staff or family members. All questions were carefully read to the participants by the investigator. The questionnaire was completed electronically by the patients. Demographic and cancer-related data were electronically collected for every patient from the medical record by the investigator.

### Questionnaire

The questionnaire consisted of four major parts. The first concerned the patients and their disease and included one question related to their performance status and another related to the highest level of education attained (less than high school, high school or college education, university education with bachelor's or master's degree and university education with doctoral degree). The second part was dedicated to assessing patients' sources of information on the COVID-19 vaccine. The first question was multiple choice with the following options: “friends, family members or neighbors,” “television, radio or newspapers,” “social media” and “scientific journals." The following question was identical to the previous one, but patients chose the major source of information from the abovementioned options. In the third part, patients were asked if they feel more vulnerable to a COVID-19 infection than the general population due to their malignancy and was a dichotomous (yes/no) question. In the last and most important part, patients answered questions about their readiness to receive an approved COVID-19 vaccine, provided that it becomes available, by choosing one of three options: (yes, no or wait). For each option, patients selected one or more answers from a multiple-choice question asking the reason behind their decision:

- If the answer was yes, patients were directed to choose between one or more of the following choices: “yes because I need the vaccine more than other people do,” “yes, similarly to other people according to international recommendations,” “yes because I am fully confident that the vaccine is effective,” “yes because I think it is well tolerated” and “yes because it does not interfere with my treatment.”
- If the answer was no, patients were directed to choose between one or more of the following choices: “no because the pandemic is a lie,” “no because the vaccine is a conspiracy to control the world,” “no because only providence determines my fate,” "no because the vaccine is not compatible with my disease and my treatment" and “no because my disease is more serious than COVID-19 and the vaccine.”
- If patients preferred to wait, they were directed to choose between one or more of the following choices: "II wait because more data are needed to verify the vaccine's efficacy," "I wait because more data are needed to verify the vaccine's tolerance" and "I wait because I need to know more about the consequences of the vaccine in other patients who have the same illness as mine.”

## Results

### Patient characteristics

A total of 114 patients were approached for recruitment between January 25, 2021 and February 12, 2021, of whom 111 were included in the study. Two patients were excluded for having benign hematologic diseases and one patient for linguistic hindrance. All patients who were approached consented to participate in the survey.

Among all patients included, there were twice more females (66.7%) than males (33.3%). The median age at the time of recruitment was 61 (23–85) years. Only 6 patients had less than high school education level (5.4%), 47 had high school or college education (42.3%) and 58 had a university education (52.2%). Almost half of the patients had a good performance status (ECOG ≤1). Most patients were on chemotherapy (87.4%). Other types of treatment were targeted therapy and immunotherapy; one patient was on hormonotherapy and bisphosphonates, and two patients were on transfusion-only for leukemia. Additional demographic and clinical characteristics are summarized in Table 1.

**Table 1.** Demographic and clinical characteristics of the patient participants (n = 111).

Patient characteristic	n (%)
Gender	
Male	37 (33.3%)
Female	74 (66.7%)
Male/female ratio	0.5/1
Age (years)	
Median (range)	61 (23–85)
Highest level of education	
Less that high school	6 (5.4%)
High school or college education	47 (42.3%)
University education with bachelor's or master's degree	53 (47.7%)
University education with doctoral degree	5 (4.5%)
ECOG performance status	
0	25 (22.5%)
1	27 (24.3%)
2	38 (34.2%)
>2	21 (19.0%)
Type of treatment	
Chemotherapy	97 (87.4%)
Targeted therapy	17 (15.3%)
Immunotherapy	12 (10.8%)
Hormonotherapy	1 (0.9%)
Bisphosphonates	1 (0.9%)
Transfusion	2 (1.8%)
Treatment setting	
Neoadjuvant	3 (2.7%)
Adjuvant	42 (37.8%)
First-line metastatic	35 (31.5%)
Second-line metastatic	6 (5.4%)
Third-line metastatic	6 (5.4%)
Hematologic malignancy	19 (17.1%)
Purpose of treatment	
Curative	60 (54.0%)
Palliative	51 (46.0%)
Life expectancy	
<1 year (but >1 month)	24 (21.6%)
>1 year	87 (78.4%)

ECOG: Eastern Cooperative Oncology Group.

**Figure 1.**
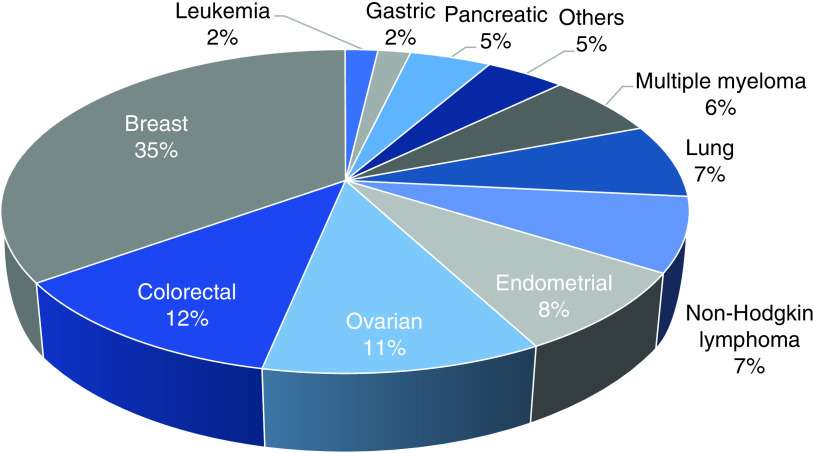
Patient distribution according to primary malignancy. Others: Cholangiocarcinoma, Hodgkin lymphoma, myelodysplastic syndrome, medulloblastoma and mesothelioma.

The most common malignancy in this sample was breast cancer (35%) followed by colorectal cancer (12%) and ovarian cancer (11%). Hematologic malignancies accounted for (17%), with the most frequent type being non-Hodgkin lymphoma (7%). Figure 1 represents the frequency of different types of malignancy in the study population.

### Patients' health beliefs regarding COVID-19

When asked if they feel more vulnerable to a COVID-19 infection than the general population because of their malignancy, 68 patients (61.3%) answered yes and 43 patients (38.7%) did not believe they were at increased risk.

### Patients' sources of information on vaccination

**Figure 2.**
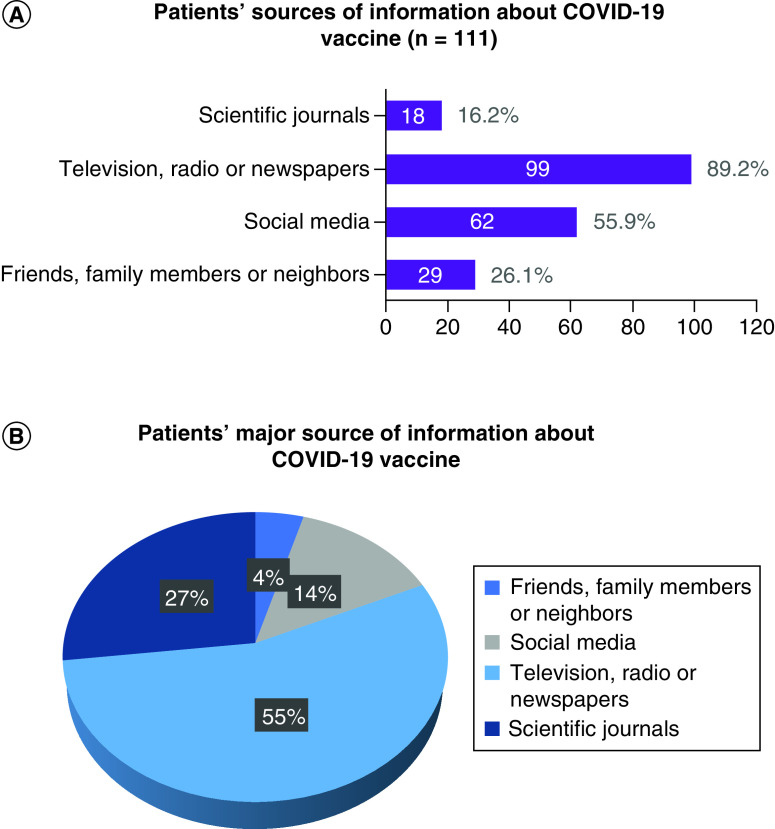
Patients' sources of information on the COVID-19 vaccines.

The investigators aimed to assess the sources of information for patients with cancer regarding COVID-19 vaccination. The participants were asked to identify all their sources of information and then choose the major source on which they rely. Analysis showed that most of the patients included learned about the vaccine from television, radio and newspapers (89.2%), and these sources also constituted the major source of information in this population (55.0%). Social media was a source of information for 55.9% of the patients; friends, family members and neighbors for 26.1% and scientific journals for 16.2%. Even though around a quarter of the patients were informed about the COVID-19 vaccine from their friends, family members or neighbors, this constituted a major source in only 4.5% of the population. These findings are detailed in Figure 2.

### Patients' willingness to get vaccinated against COVID-19

**Figure 3.**
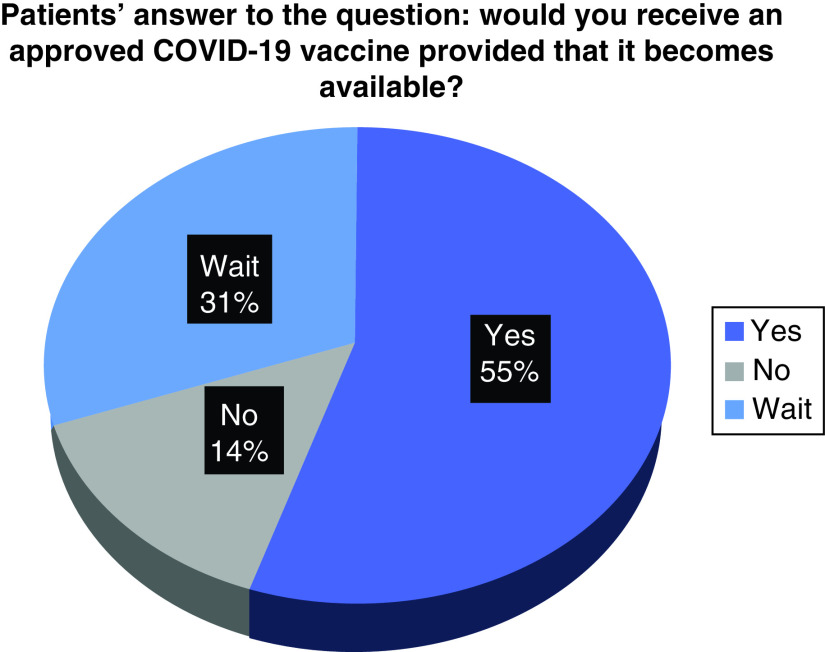
Patients' readiness to receive an approved COVID-19 vaccine.

To assess COVID-19 vaccine acceptability among patients with cancer, the participants responded to a question that asks if they would receive an approved COVID-19 vaccine whenever it becomes available. A majority of patients were ready to be vaccinated (55.0%) and 14.4% refused to get the vaccine. The remainder (30.6%) hesitated and stated that they would wait and rethink before making their decision (Figure 3).

**Figure 4.**
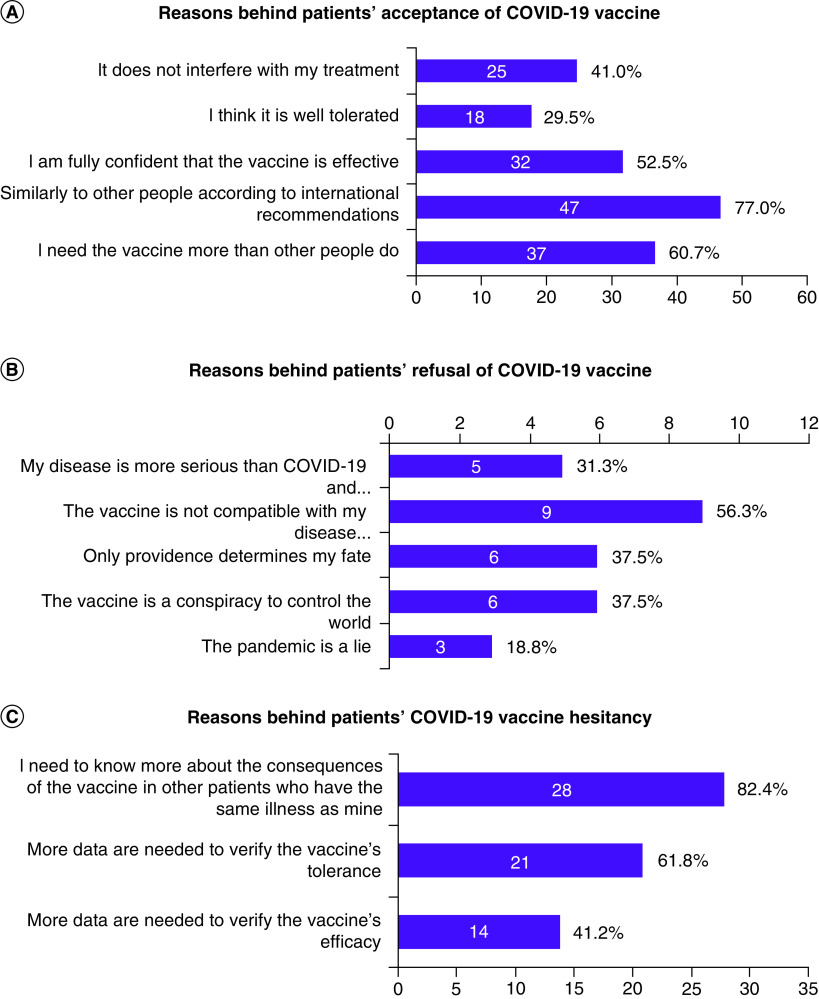
Reasons behind patients' decision on receiving the COVID-19 vaccine.

Among patients who accepted the vaccination, the main reasons for this decision were that their need for the vaccine was similar to other people according to international recommendations (77%), that they need the vaccine more than other people do (60.7%) or because it does not interfere with their treatment (41%). The main reason for refusal of the vaccine was that it is not compatible with patients' disease or treatment (56.3%), while the main reason behind vaccine hesitancy was participants' desire to know more about the consequences of the vaccine in other patients with cancer (82.4%). Figure 4 shows the reasons that drove patients' decisions on getting the vaccine in this population.

The investigators aimed to determine whether patients' perceptions of their health status, should they be infected with COVID-19, would influence their acceptance of vaccination. The acceptance rate among patients who considered themselves more vulnerable to COVID-19 was higher than in the population who did not consider themselves at higher risk (64.7% vs 39.5%, respectively). Of note, patients hesitated to take the vaccine and postponed their decision in both groups (17 patients). They accounted for 25.0% of the population who self-evaluated as more vulnerable to COVID-19 and 39.5% of the population that did not consider themselves more vulnerable.

## Discussion

Vaccine hesitancy in the time of COVID-19 has been identified as a major concern limiting healthcare professionals and authorities in their efforts to contain the pandemic [13,14]. This issue has been explored in various populations around the world, and the vaccine acceptance rate differed from one country to another. A nationwide online survey conducted in China concluded that 28.7% of the population would definitely get the vaccine, and 54.6% probably would [15]. In Italy, a survey was conducted among university students and concluded that around 14% of participants had a low intention to vaccinate (either would not vaccinate or were not sure) [16]. Although the assessment of vaccine acceptability according to educational level was not a primary objective in the present study, a higher proportion of patients with university education had a low intention to vaccinate (39.7%), of whom only 4 patients (6.9% of all patients with university education) refused the vaccine outright. In a French population, around 26% of participants in an online survey conducted before the availability of vaccines refused vaccination [17]. In the United States, vaccine acceptance rates vary between 57% and 69% depending on the targeted population [18–20]. A survey conducted in an Egyptian population, another Arab country that might share some sociocultural characteristics with Lebanon, has identified 73% of participants willing to take the vaccine once it becomes available, which is a higher rate than in the current study population (55%). However, that survey was not restricted to patients with cancer, but addressed the general population [21].

The current study assessed patients' readiness to receive the vaccine against COVID-19 in a Lebanese population. To our knowledge, this is the first survey of its kind conducted on patients with cancer. This is important to highlight due to the vulnerability of this population along with its exclusion from the phase III clinical trials that lead to the EUA for COVID-19 vaccines. Since patients with cancer have specific physical and psychological health considerations, the results cannot be compared with those of other studies conducted in the general population.

Other limitations of this study restrain its results to our population and prevent generalized conclusions. The survey was conducted in a single tertiary hospital where patients are not representative of the general population of patients with cancer. Moreover, when patient recruitment was initiated, a larger number was planned to be included. However, when the vaccine became available, we opted to end the recruitment to avoid bias. Therefore, the small sample size of our population constitutes another limitation. Although significant conclusions could not be established, this study provides insight into how patients with cancer perceive the COVID-19 vaccine and what is needed to improve vaccine acceptance.

A survey previously conducted in the same institution, at the same-day unit concluded that most of the patients insisted on getting their scheduled anticancer treatment without any delay despite their fear of exposure to COVID-19 in the hospital setting [22]. In the present study, more than half of patients expressed their fear of COVID-19 and considered themselves vulnerable. Vaccination is the safest way to protect these patients without preventing them from high-quality cancer care.

At this time, when vaccination is not sufficient to provide immunization to the entire population, especially in countries where resources are limited, prioritization guidelines have been proposed by scientific societies. For instance, the ACIP of the CDC recommends that patients with high-risk conditions be offered the vaccine during the first phase, after healthcare workers and people who are 75 years of age or older [9]. Almost a year after declaring COVID-19 a pandemic, we have sufficient data to support the vulnerability of patients with cancer to the virus and the high risk of severe complications, especially in those with hematologic malignancies. Therefore, these patients must be included in the group of people with high-risk conditions and prioritized for vaccination [23].

## Conclusion

Vaccine hesitancy has been a well-known phenomenon throughout scientific history. It is not surprising that some people remain skeptical, particularly when it is related to a novel virus that caused a global pandemic. Patients with cancer are not only vulnerable to COVID-19, but also to misinformation and misconception, especially since they are usually excluded from vaccine trials. Despite the scarce data available for this population and in the absence of enough resources, all individuals with active or prior cancer who do not have a contraindication should undergo vaccination as early as possible to prevent COVID-19 infection. We encourage all physicians, particularly in Lebanon, to enlighten their patients with accurate information regarding the vaccine and its importance in their specific case.

**Summary points**

- The COVID-19 pandemic has created a significant impact on the health of patients with cancer and recent efforts have been directed toward vaccination.
- Although patients with cancer were excluded from most of the pivotal trials that lead to the approval of the vaccines, major health and cancer authorities recommend prioritizing cancer populations in the vaccination process.
- A survey was conducted in an oncology ward among patients with solid and hematologic malignancies; 111 patients were asked to complete a questionnaire assessing their knowledge about the vaccine, their acceptance of vaccination and the reasons for their decision.
- The population was characterized by a female predominance with a preponderance of breast and gynecological malignancies, a median age of 61 (23–85) years, a level of education that is at least a high school or college in 90% of the patients and an acceptable ECOG performance status.
- Around 60% of patients with cancer considered themselves more vulnerable to COVID-19 and its complications than the general population.
- Patients acquired their knowledge about the vaccine mainly from television, radio and newspapers. Other sources of information were social media, friends and family members, and scientific journals to a lesser degree.
- A majority (55%) of patients were ready to get the COVID-19 vaccine when it became available. The main reason for this decision was the adherence to recommendations, similar to the general population. Another major reason for vaccine acceptance was that patients with cancer felt they needed the vaccine more than other patients because of their health condition.
- A total of 31% of \patients opted to wait before getting the vaccine. The major reason for vaccine hesitancy was the need for more knowledge about the consequences of the vaccine on other patients with cancer.
- Only 14% of the patients refused the vaccine outright, and this decision was mainly driven by the belief that the vaccine would not be compatible with their illness or treatment.
- Although the acceptance rate of the COVID-19 vaccine was satisfactory in this institution, better communication of information is needed to decrease vaccine hesitancy.
